# 3D Tortuosity and Diffusion Characterization in the Human Mineralized Collagen Fibril Using a Random Walk Model

**DOI:** 10.3390/bioengineering10050558

**Published:** 2023-05-07

**Authors:** Fabiano Bini, Andrada Pica, Andrea Marinozzi, Franco Marinozzi

**Affiliations:** 1 Department of Mechanical and Aerospace Engineering, “Sapienza” University of Rome, Via Eudossiana, 18, 00184 Rome, Italy; fabiano.bini@uniroma1.it (F.B.); andrada.pica@uniroma1.it (A.P.); 2 Department of Biomedical Sciences, University of Sassari, Viale San Pietro, 43/B, 07100 Sassari, Italy; 3 Research Unit of Orthopaedic and Trauma Surgery, Fondazione Policlinico Universitario Campus Bio-Medico, Via Alvaro del Portillo, 200, 00128 Rome, Italy; a.marinozzi@unicampus.it; 4 Research Unit of Orthopaedic and Trauma Surgery, Department of Medicine and Surgery, Università Campus Bio-Medico di Roma, Via Alvaro del Portillo, 21, 00128 Rome, Italy

**Keywords:** mineralized collagen fibril, diffusivity, tortuosity, random walk

## Abstract

Bone tissue is mainly composed at the nanoscale of apatite minerals, collagen molecules and water that form the mineralized collagen fibril (MCF). In this work, we developed a 3D random walk model to investigate the influence of bone nanostructure on water diffusion. We computed 1000 random walk trajectories of water molecules within the MCF geometric model. An important parameter to analyse transport behaviour in porous media is tortuosity, computed as the ratio between the effective path length and the straight-line distance between initial and final points. The diffusion coefficient is determined from the linear fit of the mean squared displacement of water molecules as a function of time. To achieve more insight into the diffusion phenomenon within MCF, we estimated the tortuosity and diffusivity at different quotes in the longitudinal direction of the model. Tortuosity is characterized by increasing values in the longitudinal direction. As expected, the diffusion coefficient decreases as tortuosity increases. Diffusivity outcomes confirm the findings achieved by experimental investigations. The computational model provides insights into the relation between the MCF structure and mass transport behaviour that may contribute to the improvement of bone-mimicking scaffolds.

## 1. Introduction

Bone is a mineralized connective tissue with a complex arrangement of structures spanning from the nano- to macro-scale. The structural organization of bone is characterized at the nanoscale by basic building blocks composed of type I collagen, apatite minerals, water and a small percentage of non-collagenous proteins and proteoglycans. Due to the complex architecture of bone tissue [1], current information concerning the correlation between the spatial organization of bone main nanocomponents and the mechanical properties, metabolic functions and transport phenomena is still elusive [2]. In fact, analysis of the cause–effect relation between bone nanostructure and mass transport properties is a topic in constant evolution [3,4,5].

The importance of diffusion for cell metabolism and the activity of proteins is well established [5]. In the context of bone nanostructure, water is responsible for the diffusion of nutrients, metabolic exchange and ion transport, and contributes to the mechanism of bone remodelling, adaptation and repair. Water is also involved in the nucleation process of apatite crystals, participating in the stabilization of mineral structure and mediating the interaction between mineral and collagen [6]. Water movement also allows bone tissue to withstand stress with less deformation [7]. The water content in bone, i.e., 10–25 wt% [8], varies with age and disease state, decreasing with increasing mineralization [9]. This can lead to an increase in bone stiffness of up to 15% for dehydrated tissue, characterized by a high mineralization condition of 40 wt% mineral, as estimated by [3].

In bone, the metabolic traffic and interchange of signalling molecules, physiological solutes and fluids are strongly dependent on the transport pathways, comprising, at the smallest hierarchical structural level, the interconnected pores within the apatite–collagen matrix [8]. The scientific literature [8,10,11] agrees that water occupies different porosity levels of bone tissue, which are nested hierarchically inside one another: collagen–apatite (10 nm), canalicular (100 nm), lacunar (up to 8 μm), vascular (50 μm), and the intertrabecular porosity (up to 1 mm).

The diffusion phenomenon within bone tissue has been studied at different length scales. At the microscale, water diffusion has been investigated in cortical bone specimens, by means of nuclear magnetic resonance [12,13]. Although this technique can non-destructively assess the water distribution in bone tissue, its resolution is limited. Subsequently, at the lacunar canalicular level, i.e., up to 8 μm, the role of fluid phase has been analysed by means of experimental and computational methods [6,14,15], especially in relation to its mechanotransduction functions. Nonetheless, a direct link between interstitial fluid flow and bone adaptation needs to be more completely established [13].

At the nanoscale porosity, initial studies have indicated that water is present as bound water. However, in the last decade, studies have introduced the hypothesis that free water may also exist at this level. Marinozzi and co-workers [16,17] performed an experimental analysis of diffusion within a single trabecula, which led to new insights for bone tissue characterization up to the length scale of its principal constituents, i.e., the collagen matrix and apatite mineral.

Within this framework, computational models have been developed as a powerful engineering tool to analyse and extend experimental findings [18]. In the literature, different in silico studies have been developed to mimic the diffusion phenomenon. With regard to the biological field, diffusion has been investigated in soft tissues such as brain [19,20] and muscle [21] tissues, and in hard mineralized tissues [22,23,24]. To date, most studies have considered 2D models, which may lead to limited predictive outcomes [19,20]. Another crucial issue lies in the difficulty of developing a method able to estimate the effects of the local variations in geometry [19].

Overall, the assessment of the diffusion coefficient can be based on empirical correlations with pore structure or on theoretical models with idealized geometry. According to [25] the influence of the structure is taken into account by means of three factors: the void space inside the porous medium, i.e., porosity, the path length that the particle has to travel in order to cross the medium, i.e., tortuosity, and the reduction of the effective flow due to the changes in the cross section of the pores, i.e., constrictivity. As highlighted by [26], the tortuosity is the main parameter that most influences the diffusion coefficient within the MCF. Tortuosity (τ) is a quantitative measure of the reduction in diffusive flux caused by the sinuous path imposed by the obstacles, compared to the straightest path in an unrestricted medium, in the direction of the flow [27]. Overall, it is influenced by the shape, arrangement and inclinations of the obstacles.

Although the existence of tortuosity and constrictivity factors in the assessment of diffusivity is recognized, a general method to extract them from experimental investigations has not been determined. The literature proposes different approaches for estimating these parameters, such as diffusion experiments [16], ultrasonic reflectivity methods [28], NMR measurements [29], analytical models [30,31] and 3D image analysis [32]. Unfortunately, any straightforward relation between the structure of the porous network and the coefficients of the diffusion process is still a topic under investigation. For instance, numerical models have been implemented to obtain a more explicit expression for tortuosity [30,31]. Overall, there are two methods presented in the literature for the calculation of tortuosity. One approach is based on the development of a model that reproduces, in detail, a region of the porous structure; then, different paths are implemented in order to determine how this structure will influence the diffusion phenomenon [31].

A second method avoids the issues related to the implementation of single paths by mimicking the diffusion process itself, e.g., the random walk approach [33]. The random walk (RW) technique represents a common method used for the investigation of mass transport dynamics, and also in biological systems [33,34,35]. The RW method describes the diffusion phenomenon in a simple manner, without the need for more complex operations [36,37], and reveals features that could be difficult to discern with other approaches. It represents, therefore, an appropriate method to investigate bone nanostructure, since experimental investigations at nanoscale are still challenging. Trajectories developed in RW models are defined as unbiased, meaning that the particle is equally likely to move in each direction, and is uncorrelated in direction, i.e., the direction of motion at a given time is independent of the directions at all preceding times [36,38].

This study aims to achieve information about the influence of the collagen–apatite structure on water diffusion at the nanoscale. The analysis is performed considering a 3D random walk model of water diffusion within the MCF, and represents an extension and improvement of a previous study [39]. In fact, we provide here a thorough analysis of the diffusion coefficient and tortuosity factor in the longitudinal direction of the fibril.

## 2. Materials and Methods

A 3-D model of the embedded apatite crystals within the collagen fibril has been developed according to the model proposed by Petruska et al. [40] and Jäger et al. [41]. The dimensions of the MCF are still debated in the literature. In this study, we considered a MCF diameter of 200 nm, following [42], while the MCF length is approximately 1000 nm [43].

Type I collagen is composed of a triple helical polypeptide chain molecule referred to as tropocollagen molecule. The collagen molecules are cylindrically shaped with an approximate diameter of 1.23 nm and a length of roughly 300 nm [44].

Apatite minerals are described mainly as platelet-like-shaped, with a low dispersion in thickness, i.e., 2–5 nm, but a wider spread in length (50–170 nm) and width (5–90 nm) [45,46]. The dimensions of apatite platelets have been obtained with random extractions from Gaussian probability distribution functions in the ranges indicated in the literature [45,46]. In Table 1, we report the average dimensions of the apatite crystals considered in this model. In the width and thickness directions, the distances between neighbouring apatite platelets, i.e., a_W_ and a_T_, respectively, are of the same order as crystal thickness, as achieved from experimental TEM observations [47]. In the longitudinal direction, the mineral platelets are arranged in a staggered pattern, with a periodicity of roughly 67 nm [40,41]. In the length direction, the distance between two consecutive apatite crystals, i.e., a_L,_ is determined according to Equation (1) [41]:

(1) $$\left(\frac{\ell +a_{L}}{2}\right)=D_{period}$$

where ℓ denotes the length of the apatite mineral, and D_period_ is the length of the axial period, i.e., D_period_ = 67 nm. Vercher-Martinez and co-workers [48] suggested parallel layers in the equatorial plane, i.e., the normal plane to the longitudinal direction.

The geometrical model represents a MCF with a mineralization degree of 32%, in agreement with previous studies [41,49]. The RW model is implemented on an MCF configuration achieved after roughly 6·10^6^ random inclinations and rotations of the platelets. We performed random displacements and rotations for each apatite platelet in order to better mimic the structure of bone tissue at the nanoscale. We started from a perfectly aligned configuration of apatite platelets and performed random perturbations to achieve inclinations for each apatite crystal, in agreement with the range available in the literature [50,51], i.e., within ±20 degrees. The method is described in detail in a previous study [42].

**Figure 1:**
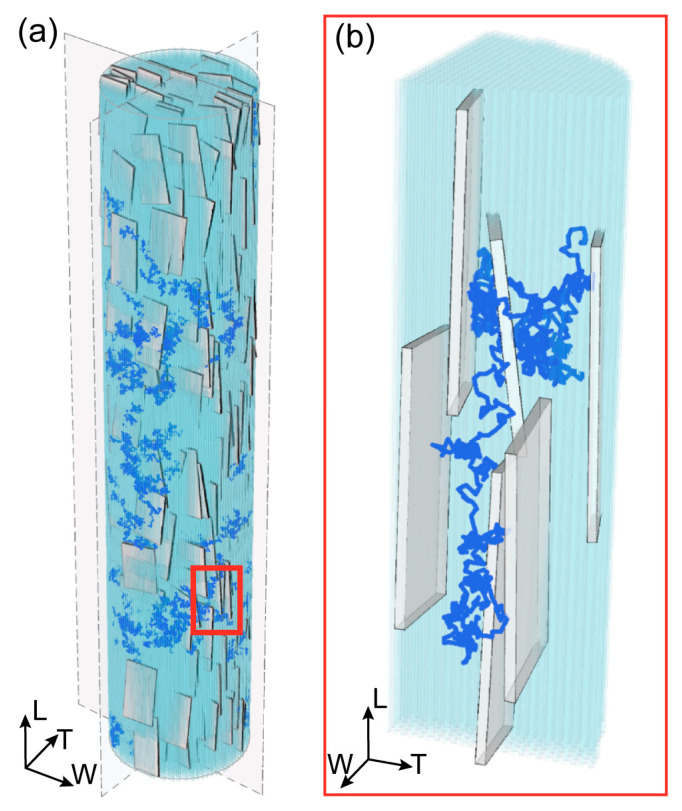
(**a**) 3D representation of the mineralized collagen fibril (MCF) and a typical trajectory of water molecule. The MCF is characterized by 32% of mineral volume fraction, and it is composed of tropocollagen (light blue) and plate-like apatite crystals (grey). The depicted configuration is obtained after roughly overall 6·10^6^ moves and rotations. (**b**) Enlarged view of a segment of the 3D random walk trajectory to highlight the path confinement within the channels created between apatite platelets and tropocollagen.

The RW model mimics the trajectory of water particles, taking into account the arrangement of the collagen–apatite structure (Figure 1). The implementation of the RW model follows a previous study by Bini and colleagues [39]. To adapt the mathematical model of RW to the spatial configuration of collagen–apatite matrix, we have assumed that apatite platelets are impermeable, and we introduced a reflection condition concerning collagen fibrils, as implemented in previous studies [33]. We also hypothesise that particle–particle interactions are negligible, since no saturation phenomenon occurs during the initial period of the diffusion process [34,37]. Moreover, in the RW model, the time interval Δt is set to 2·10^−10^ s, so as to ensure that the length of each displacement is smaller than the minimum dimensions of the structural obstacles, i.e., the cross-section of tropocollagen molecules [34,39]. In fact, the displacement δ_j_, in each coordinate direction *j*, i.e., *j* = W, T, L, is sampled from a Gaussian distribution with zero mean and standard deviation, calculated as follows:

(2) $$\sigma =\sqrt{2\cdot D_{0}\cdot ∆t}$$

where D_0_ = 2.66·10^−9^ m^2^·s^−1^ is the water diffusivity in a homogeneous medium at 27 °C [52]. This requirement was implemented to avoid the water particles skipping the obstacles, i.e., collagen fibrils and apatite crystals, which would have led to a distorted estimation of the diffusion coefficient.

The RW simulation starts by randomly positioning the water particles in the equatorial plane at the lower extremity of the MCF, i.e., coordinate L = 0 nm. Then, the particle randomly selects the coordinates of the next position. If the particle does not encounter any obstacle, it moves to the new position; otherwise, the particle stays at the current location and new coordinates are extracted randomly. This random walking procedure is repeated until the particle reaches the upper extremity of the MCF. According to the method proposed by [37], the diffusion coefficient is proportional to the slope of the linear fit of the mean square displacement (MSD) of the water particle versus time (τ).

(3) $$D=\frac{MSD(\tau )}{2\cdot d_{S}\cdot \tau }$$

where d_S_ = 3 is the system dimension. Thus, for 3D models, D equals one-sixth of the slope of the linear fit of the particle MSD as a function of τ. The MSD of the single trajectory (MSD_i_) is calculated as follows [53]:

(4) $$MSD_{i}\left(n\tau \right)=\frac{1}{N-n}\cdot \sum_{j=0}^{N-n}{\left[\vec{r}\left(j\tau +n\tau \right)-\vec{r}\left(j\tau \right)\right]}^{2}$$

where $\vec{r}$(jτ), and $\vec{r}$(jτ + nτ), are the particle coordinates at time (jτ) and (nτ + jτ), respectively, and n is the time lag, i.e., n = 1, 2, …, N − 1.

The least-squared line fit of the single MSD curve (MSD_i_) is expressed by Equation (5) [54]:

(5) $$MSD_{i}=\beta_{0}+\beta_{1}\cdot t$$

where β_0_, β_1_ are the intercept and the slope of the linear fit. The diffusion coefficient (D_i_) of the individual trajectories is obtained from Equations (3) and (5):

(6) $$D_{i}={\frac{1}{6}\beta }_{1}$$

In the available literature concerning the 3D random walk method applied to biological tissues, the number of trajectories is extremely variable, including analyses based on 30 [55], 100 [56] or 350 [33] trajectories. Saxton [53] highlighted that the numerical values of the diffusion coefficient are reliable when considering a number of trajectories (N_T_) greater than 100. In this study, we considered 1000 trajectories.

Tortuosity is an important parameter in the analysis of the diffusion phenomenon. Here, the tortuosity factor is evaluated using the random walk simulations. In this context, tortuosity is a measure of the diffusive nature of the random walk in relation to the complexity of the MCF anisotropic structure. The tortuosity of the single trajectory is defined as the ratio between the effective length of the particle paths and the Euclidean distance between the initial and final points [27]:

(7) $$\begin{array}{l} {Tortuosity}_{i}=\frac{Effective\;Length\;(i)}{Euclidean\;distance\;(i)}=\\ =\frac{\sum_{j=0}^{N-1}\sqrt{{\left(r_{W}\left(j\tau +\tau \right)-r_{W}(j\tau \right))}^{2}+{\left(r_{T}\left(j\tau +\tau \right)-r_{T}(j\tau \right))}^{2}+{\left(r_{L}\left(j\tau +\tau \right)-r_{L}(j\tau \right))}^{2}}}{\sqrt{{\left(r_{Wend}-r_{Wstart}\right)}^{2}+{\left(r_{Tend}-r_{Tstart}\right)}^{2}+{\left(r_{Lend}-r_{Lstart}\right)}^{2}}} \end{array}$$

where r_W_ (jτ + τ), r_T_ (jτ + τ), r_L_ (jτ + τ) are the coordinates in direction W, T, L of the particle position at time (jτ + τ), and r_W_ (jτ), r_T_ (jτ), r_L_ (jτ) of the particle position at time (jτ). The terms r_W start_, r_T start_ r_L start_ represent the coordinates of the initial point of the trajectory and r_W end_, r_T end_ r_L end_ are the coordinates of the final point of the path.

Since the MCF structure is characterized by high complexity, in this study, we aim to provide an analysis of the variation of tortuosity and diffusivity along the MCF. We considered 7 reference quotes in the L direction, with a step equal to the length of the MCF building block, i.e., 134 nm, in accordance with [40,41].

In order to analyse the influence of the structure, we considered sub-trajectories of the random paths that span the MCF, each starting from the inferior extremity, i.e., L = 0 nm, and reaching the equatorial planes defined by the reference quotes considered in the L direction. For each sub-trajectory, we calculated the diffusion coefficient and the tortuosity by means of Equation (6) and Equation (7), respectively.

Moreover, for each reference quote, we estimated the average tortuosity and average diffusivity from all sub-trajectories, found using the following equations:

(8) $$\begin{array}{l} Tortuosity=\frac{1}{N_{sT}}\sum_{i=1}^{N_{sT}}{Tortuosity}_{i}\\ \;=\frac{1}{N_{sT}}\sum_{i=1}^{N_{sT}}\frac{\sum_{j=0}^{N-1}\sqrt{{\left(r_{W}\left(j\tau +\tau \right)-r_{W}(j\tau \right))}^{2}+{\left(r_{T}\left(j\tau +\tau \right)-r_{T}(j\tau \right))}^{2}+{\left(r_{L}\left(j\tau +\tau \right)-r_{L}(j\tau \right))}^{2}}}{\sqrt{{\left(r_{Wend}-r_{Wstart}\right)}^{2}+{\left(r_{Tend}-r_{Tstart}\right)}^{2}+{\left(r_{Lend}-r_{Lstart}\right)}^{2}}} \end{array}$$

(9) $$D=\frac{1}{N_{sT}}\sum_{i=1}^{N_{sT}}D_{i}$$

where N_sT_ is the number of sub-trajectories.

## 3. Results

**Figure 2:**
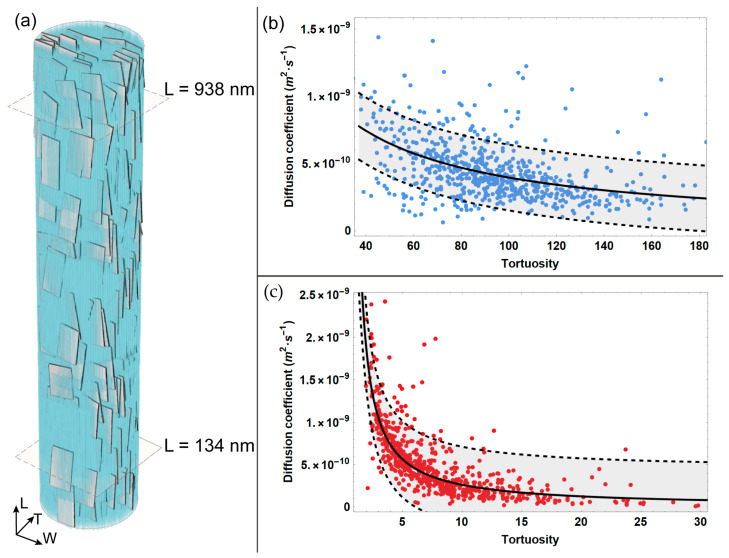
Diffusion coefficient achieved from MSD data analysis versus tortuosity for two reference quotes (**a**) that are characterized by minimum (**c**) and maximum (**b**) values of tortuosity. The light grey bands in (**b**,**c**) represent the confidence interval at 95%.

In this study, we developed a computational model simulating the 3D random walk of water particles in the regions confined by apatite platelets and tropocollagen molecules, in order to provide a thorough analysis of the diffusion phenomenon along the MCF. The effect of the structural organization of apatite minerals and collagen on the diffusivity is highlighted for different reference quotes in the longitudinal direction. We estimated the tortuosity and the diffusivity for random walk sub-trajectories that reach the aforementioned reference quotes. In Figure 2, we report the diffusion coefficient as a function of the tortuosity, and the corresponding fit for two extreme cases, characterized by the lowest (Figure 2c) and highest (Figure 2b) values of tortuosity. We also calculated the 95% confidence interval for the diffusion coefficient values estimated for the two quotes.

According to its definition (Equation (7)), the tortuosity is characterized by values greater than 1. From Figure 2, it emerges that for the reference quote sited at the lower extremity of the MCF, i.e., L = 134 nm, the tortuosity assumes modest values, with a maximum of 30. However, it can be noticed that at this reference quote, the majority of sub-trajectories are characterized by low tortuosity, with values up to 5. Conversely, at the upper position of the MCF, i.e., L = 938 nm, the maximum of tortuosity increases up to six times with respect to the maximum value estimated at the MCF lower extremity, reaching values up to 180. Differing from quote L = 134 nm, at the upper quote L = 938 nm, the majority of sub-trajectories span a wider interval of values, i.e., from 40 to 160.

Moreover, the analysis of the outcomes reported in Figure 2 shows that the diffusion coefficient is inversely proportional to the tortuosity. For instance, Figure 2c highlights that for lower values of tortuosity, the diffusivity is characterized by a wider interval of values, spanning from roughly 4.5·10^−10^ (m^2^·s^−1^) to 2.5·10^−9^ (m^2^·s^−1^). Specifically, a significant variation in the diffusion coefficient is estimated to correspond with the modest values of tortuosity, i.e., up to 5, while for higher values of tortuosity, the decrease in the diffusivity coefficient is characterized by a smooth slope.

Conversely, when considering the longer sub-trajectories that reach the upper quote and are thus characterized by higher tortuosity (Figure 2b), the diffusivity interval is narrower, i.e., from 1·10^−10^(m^2^·s^−1^) to 1.5·10^−9^ (m^2^·s^−1^). Consequently, at the upper reference quote L = 938 nm, the diminution of the diffusion coefficient for increasing tortuosity can be described as a modest slope.

**Figure 3:**
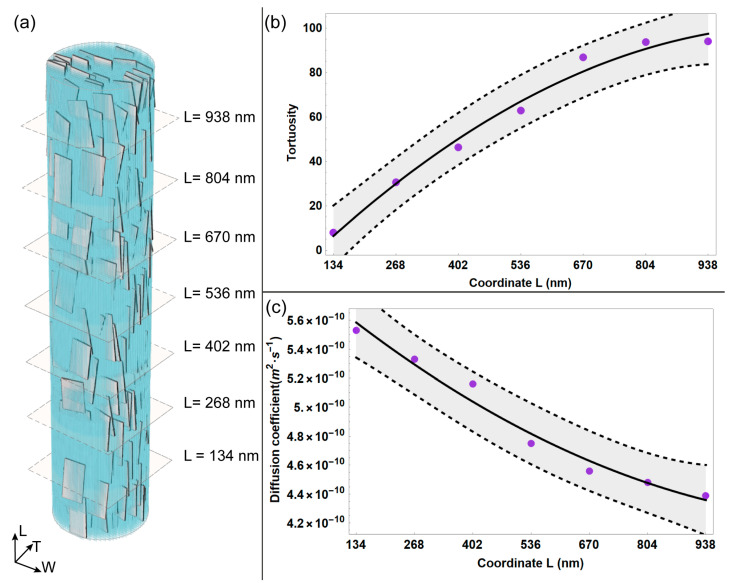
(**a**) Equatorial planes depicted correspondingto each reference plane considered in the longitudinal direction of the MCF. Average tortuosity (**b**) and average diffusion coefficient (**c**) are represented as a function of the reference quote in the longitudinal (L) direction. The light grey bands in (**b**,**c**) represent the confidence interval at 95%.

We further developed the analysis of local diffusion phenomenon by calculating the mean values of diffusivity and tortuosity for each reference quote considered (Figure 3), applying Equation (8) and Equation (9), respectively. Overall, the average tortuosity shows an increasing trend for random walk sub-trajectories of a higher extension. It can be noticed that for sub-trajectories that reach the upper reference quote L = 938 nm, the average tortuosity increases noticeably, roughly ten times with respect to the case characterized by shortest sub-trajectories, i.e., reference quote L = 134 nm. It is worth pointing out that in this study, tortuosity is an indicator of the complexity of the random walk path of the water particle, which is influenced by the spatial arrangement of the apatite platelets and the collagen matrix. As expected, for sub-trajectories that span roughly the whole length of MCF, the average tortuosity is characterized by higher values due to the increased occurrence of obstacles, i.e., mineral platelets and tropocollagen, along the MCF.

Consequently, the average diffusion coefficient is characterized by a decreasing trend with the increasing length of the random walk sub-trajectories. Specifically, the diffusivity diminishes for longer sub-trajectories (by roughly 20% with respect to the value estimated for the reference quote characterized by shortest sub-trajectories, i.e., L = 134 nm).

## 4. Discussion

Water plays a fundamental role in the provision of nutrients and the interaction between apatite crystals and protein constituents [6], enhancing the strength of the mineral phase within the MCF. An impairment of the diffusion process may also affect the bone-remodelling process [11], since mass transport contributes actively to the signalling functions that are involved in tissue growth.

Experimental analysis of the diffusion phenomenon at the bone nanoscale is still limited in the literature.

Thus, in this study, we used computational modelling to provide a more detailed analysis of the water diffusion phenomenon within the entire MCF. In fact, computational modelling can simulate the physical, chemical and biological aspects of the real system and study their interactions from a cause–effect approach. Moreover, the model developed here allows us to analyse locally, at different quotes in the longitudinal direction, how the random walk of water particles is influenced by the structural constraints derived from the spatial arrangement of apatite platelets and the collagen matrix.

It should be noted that previous studies [26,50] have investigated diffusivity, mostly considering the building blocks of the MCF. The potentiality of the RW technique with regard to the diffusion coefficient has been observed in a former study [39], in which RW simulations were applied to investigate the influence of the mineralization degree on the diffusion coefficient. In the study of Bini and colleagues [39], the computational model is used to investigate different mineral contents, i.e., low (7%), intermediate (32%), and high (42%) volume fractions. The outcomes of [39] highlight that an increase in the mineral content within the MCF leads to a diminution of the diffusion coefficient. In addition to the results presented in [39], we focused here on the local characterization of the diffusivity within the MCF characterized by 32% mineralization, which represents a physiological value of the MCF mineral content [26,41,42,49]. Moreover, the computational model allowed us to perform an analysis of the tortuosity of water particles’ trajectories, and their local variation within the entire MCF.

The collagen–apatite structure influences both tortuosity and the diffusion coefficient. The diffusivity is significantly lower than the free water diffusion coefficient because of the steric constraints of the apatite mineral configuration and collagen. Overall, the diffusion coefficient is characterized by an order of magnitude of 10^−10^ (m^2^·s^−1^), in good agreement with previous outcomes from experimental [16] and computational investigations [22]. Marinozzi et al. [16] estimated a diffusion coefficient of (4.12 ± 0.8) 10^−10^ (m^2^·s^−1^) from experimental measurement during the swelling of a single human trabeculae. Lemaire et al. [22] investigated using molecular dynamics water flow within the collagen–apatite network and achieved a diffusivity value of 8·10^−10^ m^2^·s^−1^. It is worth pointing out that studies based on the molecular dynamics method take into account a limited region of the MCF due to the high computational cost of this technique. Conversely, the RW model presented here considers the entire MCF in its assessment of water diffusion coefficient. The results are also in good agreement with previous studies that have analysed the diffusion phenomenon in bone tissue from animal samples. For instance, Gul-E-Noor et al. [57] reported a water diffusivity of 6.2 ± 0.7·10^−10^ m^2^·s^−1^ from measurements in goat cortical bone. In the work of Wang et al. [58], the fluorescein diffusion coefficient at the lacunar-canalicular level of mouse bone samples was determined to be 3.3 ± 0.6·10^−10^ m^2^·s^−1^.

Moreover, it is worth pointing out that local analysis of the tortuosity and diffusivity at different quotes in the longitudinal direction of the MCF allows us to achieve a first assessment of the obstructive effect related to apatite platelets and the collagen matrix. As expected, longer sub-trajectories are characterized by higher values of tortuosity and a lower diffusion coefficient, since these parameters are affected by the spatial arrangement of the MCF main nanocomponents. This study focused on the analysis of the random trajectories developed within the MCF. However, a more detailed analysis and quantification of the obstructive factor that characterizes the nanoconfined structure of the MCF will be the subject of future work.

The outcomes that emerged from this analysis related to the mass transport behaviour within the MCF nanostructure may facilitate the development of appropriate synthetic systems. Biomimetic scaffolds for bone should match the properties of the original tissue, such as osteoinductivity, osteoconductivity, biocompatibility, suitable porosity and pore interconnectivity, in order to allow the diffusion of cells, growth factors, nutrients, and waste products [59]. In addition, the appropriate architecture of the scaffold should be developed in order to meet the mechanical demand of the tissue environment. Application of nanotechnology [60], high-performance biomaterials [61,62,63] and computational models [24,26,39] can contribute to avoiding cell death due to insufficient nutrient transport, inadequate integration of the regenerated tissue with the surrounding native tissue, and mismatching of scaffold properties with respect to the host tissue.

Random walk is a mathematical model commonly used to describe the diffusion of molecules or particles in biological tissues that allows us to investigate the influence of structural hindrance on diffusivity. However, some limitations should be highlighted. We considered an ideal geometry for tropocollagen and apatite crystals. Tropocollagen is modelled with long cylinders, while mineral platelets are presented as regular, thin parallelepipeds. This strategy allowed us to represent the relevant characteristics of bone nanostructure whilst also maintaining a low computational cost. In this work, we assumed that the water particles start their path from the lower extremity of the MCF, i.e., L = 0 nm. A future study will also consider the propagation of water particles from the lateral surface of the MCF in order to mimic immersion in water [16].

## 5. Conclusions

The properties of bone are closely related to its hierarchical structure. The interplay between the main constituents of bone at the nanoscale affects its structural characteristics and its mass transport properties. Information concerning the behaviour and interactions of water at the nanostructure level of bone tissue is limited in the literature. In this study, we analysed tortuosity and diffusivity along the single MCF in order to predict the local variation of these parameters. The agreement between the numerical values of the diffusion coefficient determined by the RW model and the literature confirms the suitability of the geometric dimensions and spatial arrangement considered for apatite platelets and collagen. In addition, the computational model provides insights into the MCF structure and the cause–effect relationship with mass transport properties, thereby enhancing our understanding of bone mineralization.

The outcomes presented here may contribute to improving the design of smart structural nanomaterials and biomimetic scaffolds. Nowadays, the optimization of bioscaffolds mimicking the hierarchical structure of bone represents a well-known challenge. The RW model can be applied in combination with experimental investigations, and could provide new insights for more accurate tissue characterization, which could be of use in controlling the mechanical and biological performance of bioscaffolds.

## Figures and Tables

**Table 1:** Average dimensions of apatite platelets.

Parameter	Mean Value
Width	41.80 nm
Thickness	3.55 nm
Length	94.51 nm
a_W_	13.19 nm
a_T_	2.35 nm
a_L_	39.49 nm

## Data Availability

The data presented in this study are available on request from the corresponding author.
